# DSOMF: A Dynamic Environment Simultaneous Localization and Mapping Technique Based on Machine Learning

**DOI:** 10.3390/s24103063

**Published:** 2024-05-11

**Authors:** Shengzhe Yue, Zhengjie Wang, Xiaoning Zhang

**Affiliations:** School of Electromechanical Engineering, Beijing Institute of Technology, Beijing 100081, China; 3120215111@bit.edu.cn (S.Y.); xnzhang@bit.edu.cn (X.Z.)

**Keywords:** dynamic scene, simultaneous localization and mapping, direct method, instance segmentation, video inpainting

## Abstract

To address the challenges of reduced localization accuracy and incomplete map construction demonstrated using classical semantic simultaneous localization and mapping (SLAM) algorithms in dynamic environments, this study introduces a dynamic scene SLAM technique that builds upon direct sparse odometry (DSO) and incorporates instance segmentation and video completion algorithms. While prioritizing the algorithm’s real-time performance, we leverage the rapid matching capabilities of Direct Sparse Odometry (DSO) to link identical dynamic objects in consecutive frames. This association is achieved through merging semantic and geometric data, thereby enhancing the matching accuracy during image tracking through the inclusion of semantic probability. Furthermore, we incorporate a loop closure module based on video inpainting algorithms into our mapping thread. This allows our algorithm to rely on the completed static background for loop closure detection, further enhancing the localization accuracy of our algorithm. The efficacy of this approach is validated using the TUM and KITTI public datasets and the unmanned platform experiment. Experimental results show that, in various dynamic scenes, our method achieves an improvement exceeding 85% in terms of localization accuracy compared with the DSO system.

## 1. Introduction

Simultaneous localization and mapping (SLAM) technology facilitates the acquisition of location and environmental information through pose estimation and map construction [1]. In recent years, visual SLAM technology, as a subject of extensive research, has advanced significantly [2]. Sophisticated visual SLAM algorithms have been developed to achieve localization precision at the centimeter level and can be used to successfully construct large-scale three-dimensional (3D) maps [3,4,5,6,7,8]. However, these advanced visual SLAM algorithms operate predominantly under the strong assumption of rigid scenes, which substantially limits their applicability to dynamic environments. 

With the development of deep learning technologies, an increasing number of researchers have integrated object detection and semantic segmentation algorithms with classical SLAM algorithms to enhance their robustness in dynamic scenarios. Dyna-SLAM [9] combines semantic segmentation algorithms with geometric methods to identify and remove dynamic objects from images. Dm-SLAM [10] utilizes instance segmentation information and optical flow data to mitigate the effects of dynamic objects in a scene. Detect-SLAM [11] employs object detection algorithms to identify dynamic feature points and uses the motion probability to convey the motion information of these points. However, these algorithms were improved using feature-based SLAM systems. When most of the selected feature points originate from dynamic objects, the localization accuracy of the SLAM algorithm can decrease significantly because of the reduced number of usable feature points. By contrast, a more flexible selection mechanism for photometric gradient points can prevent the selection of points from being overly concentrated. Moreover, the frame-to-frame matching speed of the SLAM algorithms based on direct methods is higher.

Direct Sparse Odometry (DSO) is a visual odometry algorithm that estimates the camera motion in a sparse and direct manner [6]. Unlike traditional feature-based methods, which extract and match key-points across images, DSO operates directly on pixel intensities. This method significantly enhances the flexibility of map point selection, effectively avoiding the decrease in positioning accuracy caused by an excessive number of feature points from dynamic objects. While DSO primarily focuses on static scenes, the authors also mentioned in his paper that direct methods have a greater advantage when dealing with dynamic objects. During our practical applications, we have also observed that DSO exhibits greater robustness compared to ORB-SLAM2 when dynamic objects are present in the environment.

Although the DSO algorithm holds significant advantages over feature-based SLAM algorithms when it comes to handling dynamic environments, its adoption for further research and improvement is limited due to its low code readability and the challenging nature of modifying its direct method-based optimization approach. In this work, we not only integrate the DSO algorithm with semantic segmentation and video inpainting algorithms but also restructure its algorithmic framework. We introduce a SLAM method tailored for rigid dynamic scenes, named DSOMF. This method employs instance segmentation and video inpainting algorithms to mitigate the impact of dynamic objects, thereby enhancing the localization precision and mapping performance of the DSO algorithm. The principal contributions of this study are as follows:

1. We refine the region segmentation approach within the DSO algorithm, accelerating the motion recognition speed of the algorithm through dynamic region segmentation and the direct selection of optical flow points.
2. We propose a method that utilizes inter-frame semantic information to identify and remove dynamic objects. This approach effectively reduces the interference caused by noise introduced by dynamic objects, enhancing the system’s robustness in dynamic environments. Furthermore, we also improve the matching accuracy of pixel intensities points by adding semantic probability, so that our algorithm can make full use of semantic information.
3. We synergize video inpainting algorithms with the map-building thread of the DSO algorithm, compensating for static background gaps caused by the removal of moving objects, thereby optimizing the map construction performance of the DSO algorithm in dynamic environments.
4. We integrate a loop closure detection module, thus rendering the DSO algorithm framework more comprehensive. Moreover, by associating loop closure detection with video inpainting algorithms, we enhance the efficiency of this module in identifying loop closures within dynamic environments.

In the following, we discuss the related studies in Section 2, providing a comprehensive introduction to its methodology in Section 3, followed by a detailed elaboration on motion object segmentation, semantic data association, motion object recognition, and static background completion, and a comparative analysis of the positioning accuracy between our algorithm and existing ones conducted using indoor and outdoor datasets in Section 4. The real-scene experiments based on the unmanned aerial vehicle and unmanned vehicle platforms is in Section 5 and we conclude the paper in Section 6.

## 2. Related Works

In recent years, SLAM algorithms for dynamic environments have received considerable attention from the research community, thus resulting in the proposal of a multitude of dynamic environment SLAM algorithms. Based on the different approaches for managing dynamic objects in the environment, the solutions reported in the literature can be categorized into two types, which are detailed in the next subsections.

### 2.1. Eliminating Dynamic Objects

In one of the SLAM algorithm types, dynamic objects in images are typically classified as outliers for removal. Facilitated using machine learning algorithms, the primary approach of such SLAM algorithms is to use semantic segmentation or object detection to identify objects in images that can potentially move. Subsequently, the motion states of these potentially movable objects are assessed using geometric methods [12,13,14]. Finally, the dynamic features of the moving objects are eliminated using an improved RANSAC algorithm [15,16,17]. Detect-SLAM [11] combines ORB-SLAM2 with the SSD object detection algorithm, where motion probabilities are used to convey the dynamic information of feature points and remove dynamic points that reach a certain threshold. DS-SLAM [18] employs the SgeNet semantic segmentation algorithm to delineate dynamic object regions and delete all feature points within an area if dynamic features are detected. Moreover, DS-SLAM builds an octree map using semantic information and filters unstable voxels using a logarithmic scoring method. RDS-SLAM [19] utilizes a Bayesian filter to identify and remove dynamic objects and has been used to design a novel semantic segmentation thread execution to reduce the effect of semantic segmentation algorithms on the real-time performance of the SLAM system. Alcantarilla et al. [20] employed scene flows to remove dynamic objects from an environment. Although these SLAM methods discard dynamic objects in images, thereby enhancing the system robustness and estimation accuracy, they also reduce the ability of the SLAM system to capture static environmental information. Consequently, some SLAM algorithms that target dynamic scenes complete an incomplete static background after removing dynamic objects. Dyna-SLAM [9] removes dynamic objects using a combination of semantic segmentation and geometric methods and then applies multi-view geometry information to color and complete the missing background.

### 2.2. Constructing Dynamic Objects

In another type of SLAM method, the SLAM algorithm is primarily integrated with moving object tracking (MOT) algorithms. Thorpe et al. [21] performed SLAM and MOT in separate threads, where the MOT thread was isolated to detect and track dynamic objects, thus ensuring the real-time performance of the SLAM system. Reddy et al. [22] not only combined MOT with structure from motion to construct maps that included both the structure of static objects and the trajectories of dynamic objects but also introduced semantic constraints to achieve more precise results of 3D reconstruction. Owing to advancements in technologies such as 3D object detection, researchers have begun using simple geometric shapes to represent dynamic objects, thus facilitating their incorporation into static maps. Salas-Moreno et al. [23] first proposed an object-specific SLAM algorithm incorporating both object and camera poses into backend optimization. Subsequently, Tateno et al. [24] combined SLAM with 3D object detection incrementally and in the real-time segmentation of a 3D scene during map reconstruction, thus enhancing the robustness of the object-recognition system. Node-SLAM [25] introduced multiple classes of learning objects and a new probabilistic and differential rendering engine to derive complete object shapes from one or more RGB-D inputs. Hosseinzadeh et al. [26] was the first to use ellipsoids to envelope dynamic objects for the parametric representation of their size and pose. Building upon this concept, researchers proposed quadric-SLAM [27], where the geometric parameters of dynamic objects were integrated into the factor graph of the SLAM system for joint optimization. Additionally, researchers introduced Dyna-SLAM II [28], where dynamic objects were enveloped using 3D object detection frameworks and proposed a formula for estimating the size and pose of dynamic objects, thus allowing the system to simultaneously estimate the camera pose, map, and trajectory of moving objects.

Taking into account the two approaches outlined above, while the concept of constructing dynamic objects is more advanced, the current target tracking and 3D object detection technologies both require significant computational resources. Furthermore, existing SLAM algorithms do not accurately calculate the speed of dynamic objects. Therefore, we still adopt the approach of excluding dynamic objects to maximize the positioning accuracy of our SLAM system in dynamic environments.

## 3. Algorithm Design

**Figure 1:**
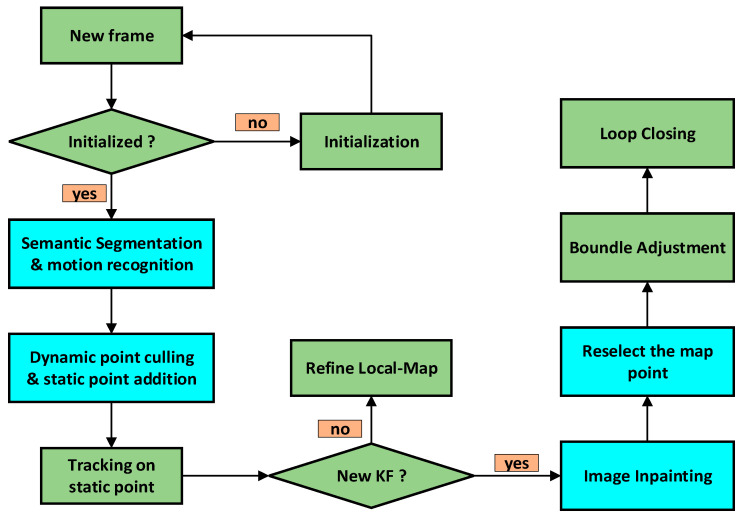
Algorithm framework.

In this section, we first introduce the overall algorithmic framework of the proposed SLAM system, followed by a detailed discussion of the methods introduced herein. This study addresses the problem of localization and mapping in dynamic environments and proposes an algorithm framework based on DSO, as illustrated in Figure 1. Within this framework, the regions highlighted in blue represent programs that have been added or modified, whereas those in green indicate the original programs.

**Figure 2:**
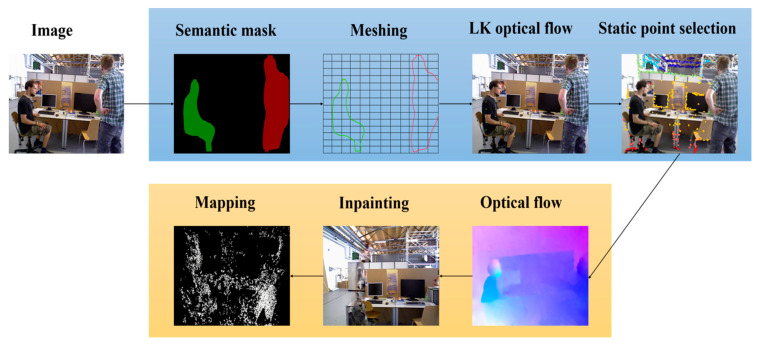
Image processing workflow (The blue box in the diagram represents the tracking thread, while the orange box represents the mapping thread).

Firstly, we employed an instance segmentation network, i.e., mask R-CNN [29], to extract all prior instances of dynamic objects. Subsequently, we utilized an enhanced PARSAC algorithm [30] for the motion recognition of dynamic object instances and for eliminating photometric gradient points within the dynamic object regions. Once elimination was completed, photometric gradient points were supplemented from static regions to increase the number of trackable points for the algorithm. After selecting the keyframes, we applied the flow-guided video completion (FGVC) algorithm [31] to replenish the static background occluded by dynamic objects in the keyframes. Finally, we re-supplemented photometric gradient points from the replenished portions of the keyframes and input them into the mapping thread for loop closing. The image processing procedure is depicted in Figure 2 below, with the tracking thread enclosed in blue and the mapping thread in orange.

### 3.1. Segmentation of Potential Dynamic Objects

To detect objects within an image that possesses the potential for motion, we employed a mask R-CNN instance segmentation network to segment the input image. The mask R-CNN network is a widely used instance segmentation technique that enables the acquisition of pixel-level semantic segmentation outcomes, along with instance-level object labels. Current semantic SLAM algorithms designed for dynamic environments typically utilize only semantic segmentation information and directly eliminate objects that are likely to move. This approach inadvertently removes objects that are potentially mobile yet stationary, thus diminishing the number of static feature points available for the algorithm and affecting the precision of SLAM localization. This study innovatively integrates semantic segmentation information with instance label data, thereby refining the identification of moving objects to the level of individual instances, to preserve as many static points in the image as possible.

**Figure 3:**
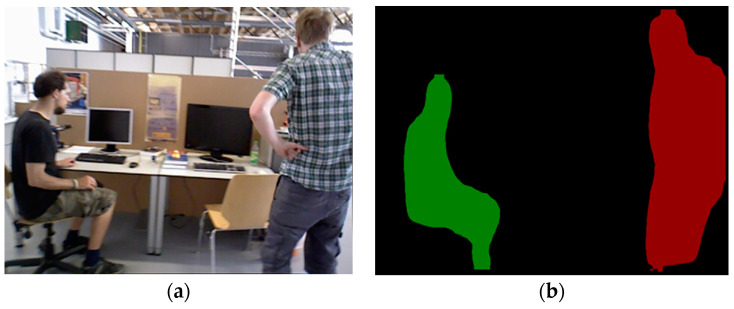
Dynamic object instance segmentation results. (**a**) Original image; (**b**) mask image.

The input for the mask R-CNN is the raw image captured using the camera, as shown in Figure 3a. In this study, the mask R-CNN network was exclusively utilized to segment the categories of objects that exhibit the potential for dynamics, including but not limited to movable humans, bicycles, cars, cats and dogs. These categories are posited to encompass the most dynamic objects likely to appear in most scenes. If additional object categories must be identified, then one can retrain the mask R-CNN network using the COCO dataset [32] and then fine-tune its weight files accordingly.

For the output results of the mask R-CNN, we assumed that the input image size was *s × h × 3*. During processing, the network generated an *s × h × w* matrix, where *w* represents the number of dynamic objects in an image. Each output channel (*c* ∈ *w*) generates a binary mask image for a specific object instance. Stacking these *w* images yielded a segmented result that encompassed all instances of dynamic objects, as illustrated in Figure 3b.

### 3.2. Region Segmentation and Data Association

**Figure 4:**
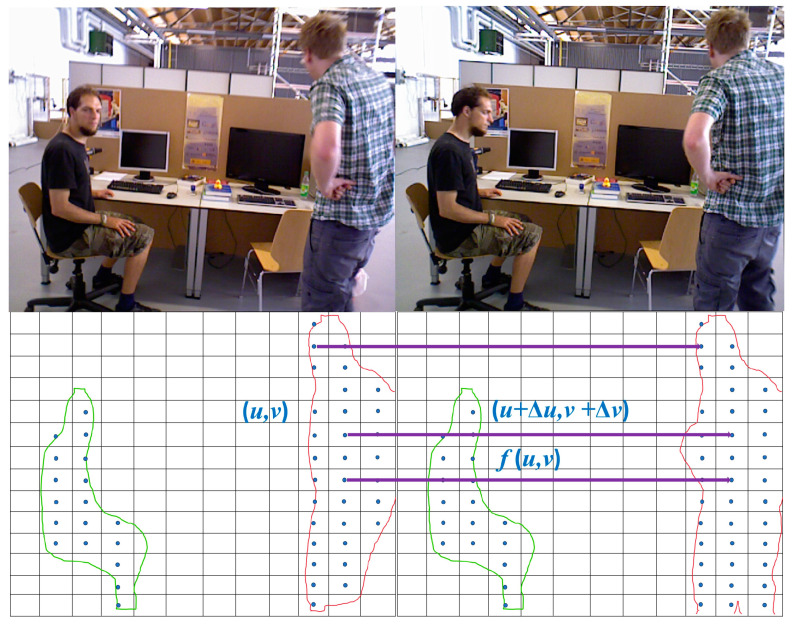
Optical flow tracking of regional centroids.

The DSO performs tracking by selecting points with photometric gradients within an image. To achieve a more uniform point distribution, the DSO segments the image into n square regions, each with a side length of *d*, and selects photometric gradient points within each region. To further conserve computational resources, this study enhances the original algorithm by redefining the partitioning of regions based on the number and size of potentially moving objects in an image, thereby facilitating motion detection. An analysis of semantic segmentation labels across multiple datasets revealed that when the labels indicating potential motion exceeded 30% of the total label count, the image was likely to contain a higher number and larger projection area of moving objects. Consequently, when the proportion of labels for potentially moving objects exceeded 30%, the image was partitioned into square regions with a side length of 6d. Otherwise, the regions were defined as having a side length of 4d. Upon completing the partitioning of regions, the central point of each region was identified, and the Kanade–Lucas–Tomasi method was applied to track their optical flow *f*: (*u*, *v*) → (*u* + Δ*u*, *v* + Δ*v*). Thus, the correspondence between the segmentation regions of the two frames was identified, as illustrated in Figure 4.

The association degree between segmentation regions of successive frames can be represented as follows:

(1) $$\varphi_{f}(C_{a}^{t-1},C_{b}^{t}|Z,f_{t-1\to t})=\frac{\sum_{k=1}^{N}\left[Loc(f(z_{i}),C_{b}^{t})\times Loc(z_{i},C_{a}^{t-1})\right]}{\sum_{k=1}^{N}Loc(z_{i},C_{a}^{t-1})},$$

where *φ_f_* denotes the association degree of regional centroids, *Z* the observation function, *z_i_* the observation point, *a* and *b* the semantic tag and $C_{a}^{t}$, $C_{b}^{t}$ the semantic label clustering of central points at time *t*. By defining the spatial association of segment clustering, we can represent the overlap between segmented regions across two frames [33]. Specifically, it can be articulated as:

(2) $$\varphi_{s}(C_{a}^{t-1},C_{b}^{t-1})=\frac{\left\|C_{a}^{t-1}\cap C_{b}^{t}\right\|}{\sum_{k=0}^{N}C_{k}^{t-1}},$$

where, *φ_s_* denotes the spatial association of segmentation clustering. By considering both the spatial and centroid associations, the probability that ($C_{a}^{t-1}$, $C_{b}^{t}$) belongs to the same category can be expressed as:

(3) $$\begin{array}{l} P=(l_{i}^{t}=j|C_{t-1},C_{t},L_{t-1},Z,f_{t-1\to t})=\\ \frac{\varphi_{s}(C_{i}^{t-1},C_{j}^{t})+\alpha \times \varphi_{f}(C_{i}^{t-1},C_{j}^{t})}{\sum_{m=0}^{N}\sum_{n=0}^{N}\left[\varphi_{s}(C_{m}^{t-1},C_{n}^{t})+\alpha \times \varphi_{f}(C_{m}^{t-1},C_{n}^{t})\right]} \end{array},$$

where, $l_{i}^{t}$ denotes the object label. Consequently, the label of $C_{i}^{t}$ can be expressed as:

(4) $$l_{i}^{t}=\arg\max P(l_{i}^{t}=j|C_{t-1},C_{t},L_{t-1},Z,f_{t-1\to t}),$$

The equation above clearly indicates, that when $l_{i}^{t}=l_{0}^{t-1}$ and *i* ≠ 0, a new object label emerges in the scene, thus incrementing the label count. Using this approach, the association between the segmentation results of potential moving objects across successive frames can be established based on both centroid matching and the overlap of segmentation regions.

### 3.3. Dynamic Object Recognition and Determination of Dynamic Regions

Because the methodology used in this study was adapted from DSO, using the photometric error-based matching approach, which is computationally efficient, may compromise the accuracy of point matching. Consequently, this study incorporates a motion recognition method based on semantic probability [34], which is expressed as follows:

(5) $$\varepsilon^{2}(x,x^{\prime }F)=\frac{{\left({\bar{x}}^{\prime }F\bar{x}\right)}^{2}}{{\left(F\bar{x}\right)}_{1}^{2}+{\left(F\bar{x}\right)}_{2}^{2}+{\left(F^{T}{\bar{x}}^{\prime }\right)}_{1}^{2}+{\left(F^{T}{\bar{x}}^{\prime }\right)}_{2}^{2}},$$

where *x* denotes the coordinates of the regional centroid in the previous frame, $x^{\prime }$ the coordinates of the regional centroid in the current frame, $\bar{x}$ the coordinates in homogeneous form, and *F* the fundamental matrix between two adjacent frames. Consequently, for each label cluster, a parameter $D=\{d_{i}|i=1,2,\cdots ,N\}$ can be obtained to evaluate whether the matching of the centroid of the cluster region adheres to geometric constraints. This can be expressed as follows:

(6) $$D(Z,C_{i}^{t},F,f_{t-1\to t})=\frac{\sum_{k=1}^{N}\left[\varepsilon^{2}(z_{k},f(z_{k}),F\times Loc(z_{k},C_{i}^{t})\right]}{\sum_{k=1}^{N}Loc(z_{k},C_{i}^{t})},$$

(7) $$d_{i}=D(Z,C_{i}^{t},F,f_{t-1\to t}),$$

When *d_i_* surpasses a specified threshold $\tau_{dyn}$, we assume that motion has occurred within the associated segmentation cluster.

After determining the motion status of the segmentation clusters, regions whose centroids are dynamic cluster points are marked as dynamic areas. Additionally, there are regions where the centroid is not a dynamic cluster point but the extremities are defined as edge areas. The dynamism of the edge areas is ascertained based on the distance from the centroid to the segmentation cluster. The distance from the centroid of an edge area to the segmentation cluster is expressed as:

(8) $$dist(x_{k},C_{j})=\min{\left\|x_{k}-x_{i}\right\|}_{2},x_{k}\in {\bar{C}}_{j},x_{j}\in C_{j},$$

In the equation, ${\bar{C}}_{j}$ represents the set of edge centroids of segmentation cluster $C_{j}$ . Therefore, the semantic probability that the edge centroid *x_k_* belongs to $C_{j}$ can be expressed using binomial logistic regression as follows:

(9) $$P=\frac{1}{\exp(-\lambda \times dist(x_{k},C_{j}))+1},$$

**Figure 5:**
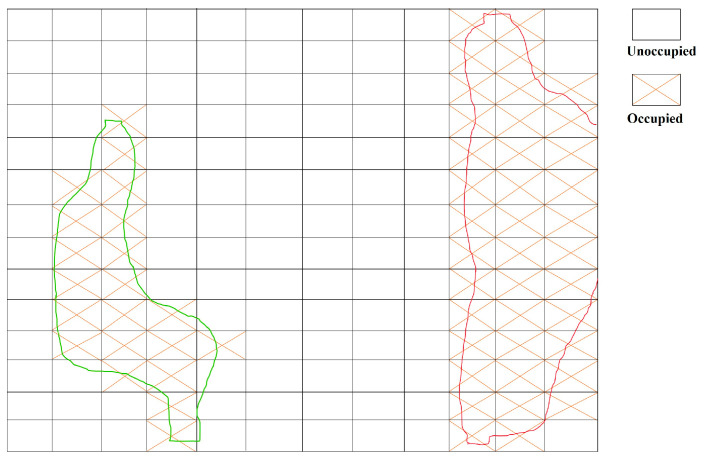
Delineation of dynamic and static regions.

When *P* exceeds *P_dyn_*, the edge area becomes dynamic. Following the determination of the dynamic areas, these regions are marked as occupied (as illustrated in Figure 5), and all photometric gradient points selected using the DSO algorithm within these areas are excluded. Subsequently, based on the proportion of dynamic areas to the total area, the threshold for selecting the photometric gradient points in the static areas is reduced appropriately to compensate for the number of points selected in unoccupied areas.

### 3.4. Completion of Static Background in Keyframes and Loop Closure

**Figure 6:**
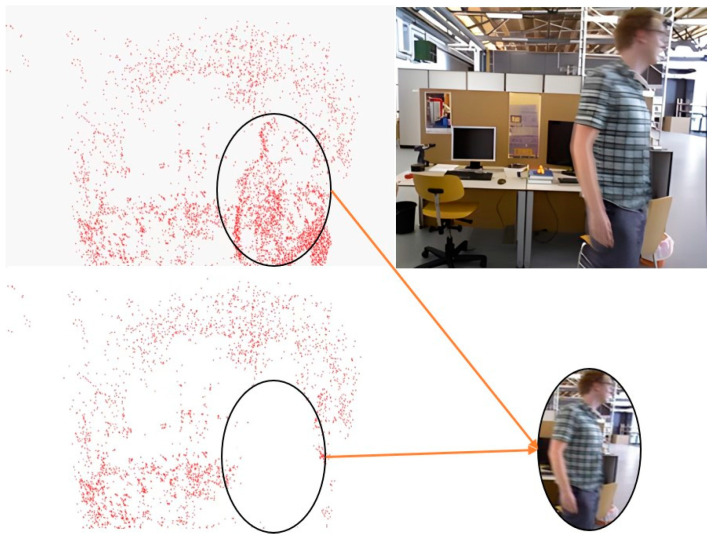
Comparative analysis of mapping outcomes pre and post dynamic object elimination.

After instance segmentation and motion-consistency verification are performed to eliminate dynamic objects, the constructed static background map may exhibit gaps, as illustrated in Figure 6. In the DSO framework, non-keyframes participate only in localization and tracking, whereas map construction relies on keyframes. Consequently, we utilized pixels from non-keyframes to fill in the static background voids caused by dynamic objects obstructing the keyframes. Thus, the keyframes can be used to synthesize more realistic static environment images after the removal of dynamic objects. This type of composite image, which includes static structures, can not only further optimize and enhance the accuracy of camera pose estimation but also contribute significantly to virtual and augmented reality applications.

**Figure 7:**
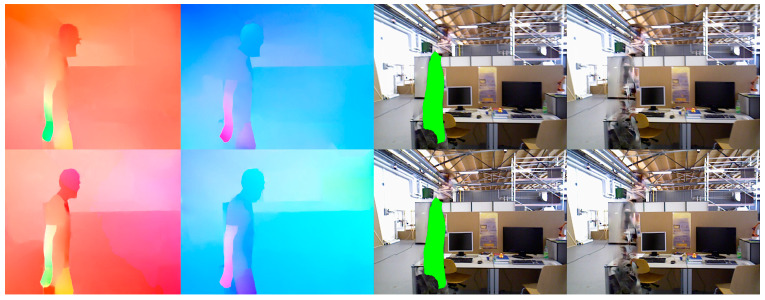
FGVC optical flow completion process.

For missing regions in an image, computing the optical flow field of the area is easier than direct pixel filling, and optical flow-guided pixel propagation can naturally maintain temporal coherence [35]. Additionally, owing to the tracking of object movement in non-key frames, their optical flow can be more readily obtained. Therefore, we adopted the FGVC algorithm, which is guided using optical flow edges. After the tracking thread identifies dynamic objects, it sends the keyframe images and dynamic object labels to the video completion thread. The FGVC video completion algorithm primarily comprises three steps (as shown in Figure 7): (1) Flow completion, where the forward and backward optical flows between adjacent frames are calculated; (2) temporal propagation, where the trajectory of the optical flow is followed to identify a set of candidate pixels for each missing pixel, and a confidence score as well as a binary validity indicator are estimated for each candidate frame; (3) fusion, where confidence-weighted averaging is employed to fuse each missing candidate pixel with at least one valid candidate pixel. Single-image completion techniques are used to fill areas devoid of candidate pixels. To ensure the effectiveness of the map completion thread, the tracking thread saves 20 frames of non-keyframes for achieving completion. After completing the missing areas, photometric gradient points are selected within the completed areas and integrated into the backend optimization thread to enhance the localization accuracy and mapping outcomes.

After completing the video inpainting, we re-selected some photometric gradient points on the incorporated static background into the mapping thread. Subsequently, we detected loop closure candidates using the Bag of Words (BoW) model, computing the Sim(3) transformation between the candidate frames and the current frame, and we applied these constraints to pose a graph optimization to enhance the overall map accuracy. This method effectively avoids failures in the Bag of Words model detection caused by occlusions from dynamic objects, thereby improving the efficiency of loop closure detection in dynamic environments.

## 4. Simulation Testing

To verify the effectiveness of the proposed method, we conducted tests using a publicly available indoor dynamic TUM dataset [36] and the outdoor dynamic KITTI dataset [37]. Because the proposed method modifies the existing stable open-source DSO algorithm, it primarily serves as a benchmark for comparison. Additionally, we compared the proposed method with a similar Dyna-SLAM algorithm, which utilizes the mask R-CNN algorithm for segmenting dynamic objects. To evaluate the effect of map completion on the localization accuracy, we used an algorithm that removes dynamic objects but does not perform map completion (named DSOM) and an algorithm that removes dynamic objects and performs map completion (named DSOMF). Additionally, we employed the absolute trajectory error (ATE) and relative pose error (RPE) for a quantitative evaluation of the algorithms’ localization accuracy. The ATE metric represents the global consistency of the trajectory, whereas the RPE metric reflects the drift in translation and rotation. All the experiments were conducted on a notebook computer equipped with an Intel Core i9-14900H CPU, RTX 4090 GPU with 16GB of graphics memory, and 64GB of RAM in a dual 32GB configuration.

### 4.1. Simulation Testing on TUM Dataset

The TUM dataset captures data for various task types in indoor environments using RGB-D cameras. Moreover, the dataset provides the ground truth for camera poses and comparison tools, thus rendering it highly suitable for SLAM researchers to evaluate the performance of their algorithms.

In this study, three sets of data from the dynamic object module of the dataset were used for simulation testing: fr3_sitting_static, fr3_walking_static, and fr3_walking_xyz. The first dataset represents a static environment, whereas the latter two are dynamic environments. The results yielded using the algorithm are shown in Table 1, where the first column lists the dataset names and the second to fourth columns are the names of the algorithms. From left to right, the second, third, and fourth columns present the root mean square error (RMSE) and standard deviation (STD), which effectively reflects the stability and robustness of the algorithm. In dynamic environments, the improvement in the localization accuracy of the proposed method relative to the other two algorithms is expressed as:

(10) $$\delta =\frac{\alpha -\beta }{\alpha }\times 100\%,$$

where *δ* represents the improvement rate, *α* the data from the other two algorithms, and *β* the data from the proposed algorithm.

Based on the data shown in Table 1, one can observe that in the fr3_sitting_static data scenario, the RMSE and STD of DSOM improved by 37% and 24%, respectively, compared with those of DSO, whereas they did not improve significantly with respect to those of Dyna-SLAM2. The RMSE and STD of DSOMF improved by 45% and 27%, respectively, compared with those of the DSO, whereas they did not improve significantly with respect to those of Dyna-SLAM2. In the fr3_walking_static data scenario, the RMSE and STD of DSOM improved by 88.5% and 65.4%, respectively, compared to DSO, and by 5.4% and 9.3%, respectively, compared with those of Dyna-SLAM. The RMSE and STD of DSOM improved by 90.6% and 71.6%, respectively, compared with those of DSO, and by 21.6% and 25.5%, respectively, compared with those of Dyna-SLAM. In the fr3_walking_xyz data scenario, the RMSE and STD of DSOM improved by 90.8% and 87.8%, respectively, compared with those of DSO, and by 10.9% and 17.5%, respectively, compared with those of Dyna-SLAM. The RMSE and STD of DSOMF improved by 92% and 89.7%, respectively, compared with those of DSO, and by 20.8% and 24.6%, respectively, compared with those of Dyna-SLAM.

**Figure 8:**
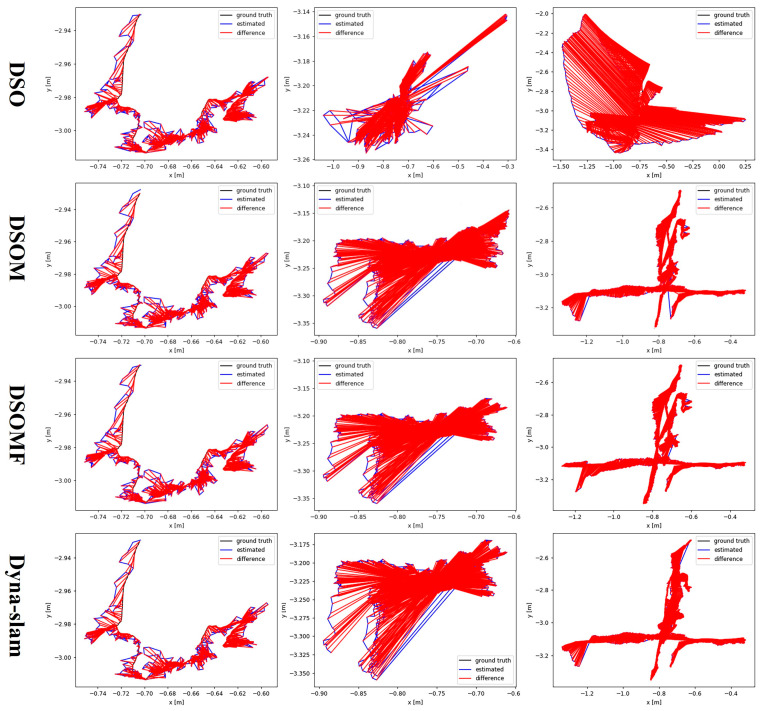
Comparison of absolute trajectory error for the camera on the TUM dataset.

To further analyze the test results, Figure 8 shows a comparison of the ATEs among DSO, Dyna-SLAM, and the methods presented here in this study, i.e., DSOM and DSOMF, across the three scenarios of fr3_sitting_static, fr3_walking_static, and fr3_walking_xyz. One can intuitively observe that as the dynamic nature of the scenes increases, the improvement effect of the methods on the localization accuracy intensifies gradually. In environments with higher dynamics, the localization effect of the methods presents a clear enhancement compared with that of DSO. However, the improvement effect of DSOMF was slightly weaker relative to that of Dyna-SLAM, which is attributed to the deteriorating image completion effect owing to the increased number of dynamic objects in the image.

To further enhance the real-time performance of our method, we drew inspiration from RDS-SLAM [19] and incorporated the selection of semantic keyframes into our algorithm. Therefore, in addition to comparing the localization accuracy, we quantitatively compared the runtime overhead and GPU memory consumption of several methods on the TUM dataset, as listed in Table 2. As shown, the per-frame processing time of DSOM was lower than that of Dyna-SLAM. After the video completion thread was incorporated, the per-frame processing time of DSOMF was slightly longer than that of Dyna-SLAM.

Additionally, Table 3 shows the time overhead of the main modules of the proposed method on the TUM dataset. Clearly, the semantic segmentation module was the most time-consuming. In this study, instance segmentation is executed as a separate, independent thread, thus allowing for the substitution with a less time-consuming instance segmentation algorithm (if necessary) to further enhance the real-time performance of the proposed method. Moreover, through efficient parallel operations, the incorporation of instance segmentation and video completion modules, in addition to DSO, results in frame rates and memory usage that are comparable to those of Dyna-SLAM.

### 4.2. Simulation Testing on KITTI Dataset

The KITTI dataset is widely recognized and utilized in the field of computer vision algorithms for testing an autonomous driving scenario. It encompasses various dynamic outdoor scenarios, including urban, rural, and highway environments. The images that captured the most dynamic objects include 15 vehicles and 30 pedestrians, thus indicating a high level of scene dynamism. Sequences 00-10 from the dataset were used to validate the DSOM and DSOMF algorithms proposed herein. The comparisons were performed made with the DSO and Dyna-SLAM algorithms using the EVO evaluation tool.

To analyze the positioning performance of DSOMF in outdoor dynamic environments, we present a comparison of the absolute pose errors between the DSOMF and DSO algorithms for dataset sequences 01, 02, 04, and 06 in Table 4. The data in the table include the corresponding RMSE, mean, max and min values. The data in the table indicate that, for sequences 01 and 02, the absolute pose error of DSOMF is significantly lower than that of DSO. For sequences 04 and 06, the absolute pose error of the method presented herein is comparable to that of DSO. This is because sequences 01 and 02 contain many dynamic vehicles and pedestrians, whereas sequences 04 and 06 contain primarily stationary vehicles.

To analyze the effect of the video completion module on the localization accuracy in outdoor environments, we present a comparison of the APEs among the DSOM, DSOMF, and Dyna-SLAM algorithms across dataset sequences 00–10 in Table 5. As shown in the table, in outdoor environments, both DSOM and DSOMF exhibited slight improvements in terms of pose-estimation accuracy across various dataset sequences compared with Dyna-SLAM. However, the inclusion of the video completion thread in DSOMF did not significantly enhance its performance compared with those of the first two methods. This is because in large outdoor scenes, dynamic objects typically occupy a relatively small area in images, and images completed thereafter fail to provide additional map information for backend optimization.

**Figure 9:**
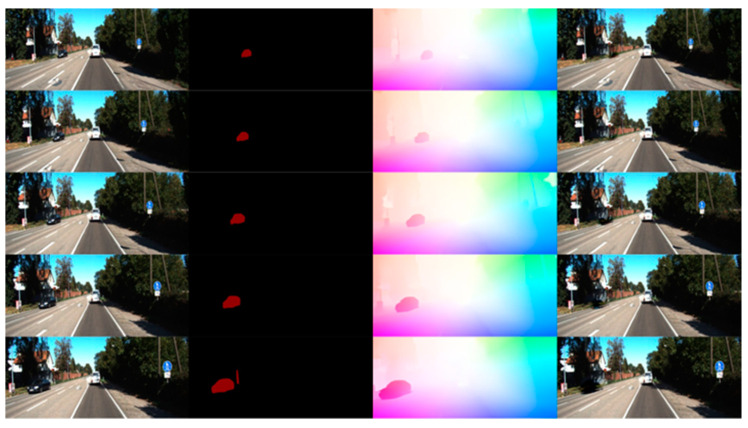
Video completion process in KITTI-04 dataset.

**Figure 10:**
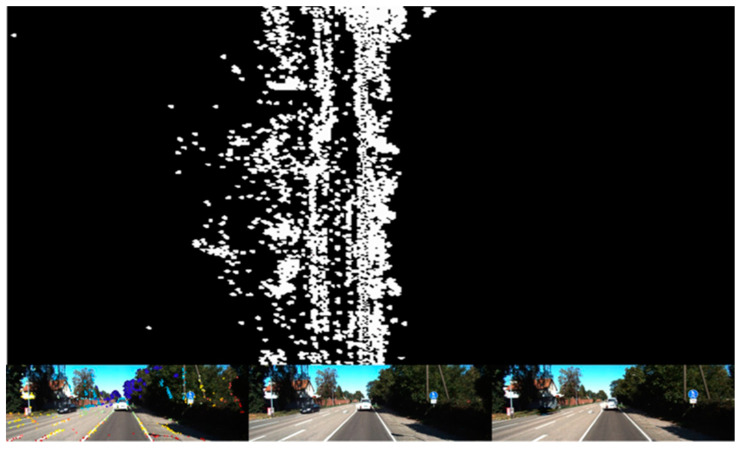
Map construction effect of DSOMF in the KITTI-04 dataset.

Figure 9 shows the process of dynamic object removal using a video completion thread in the KITTI-04 dataset. In this figure, a white vehicle moving at a constant speed in the same direction is identified as a static object, whereas a black vehicle moving in the opposite direction is recognized as a dynamic object and thus removed and completed. The map-construction effect, as presented in Figure 10, shows that DSOMF excluded the interference of the black vehicle during map construction, thus resulting in a complete, linear road point cloud map.

## 5. Unmanned Platform Experiment

### 5.1. Drone Experiment

**Figure 11:**
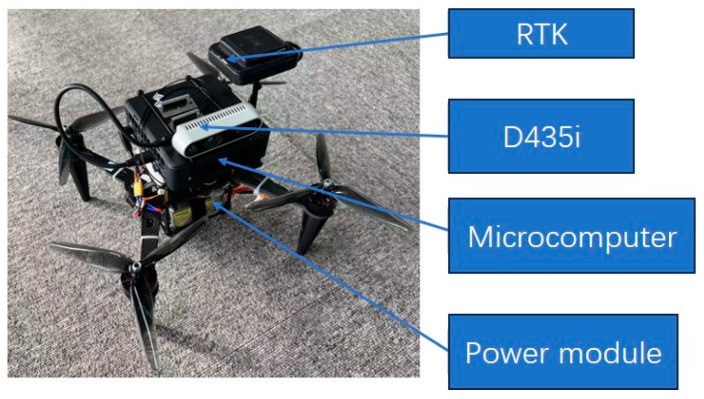
Unmanned flight platform SLAM algorithm test system.

Given the absence of distinct loops in the datasets utilized above, to verify the effectiveness of the loop closure detection module, we conducted real-world loop closure detection experiments using the quadcopter drone depicted in Figure 11. The drone is equipped with an Intel Realsense D435 image sensor (Santa Clara, CA, USA) and a micro-computer developed on the Jetson AGX Orin platform (2048-core NVIDIA Ampere architecture GPU (Santa Clara, CA, USA) featuring 64 Tensor Cores, 12-core ARM Cortex-A78AE v8.2 64-bit CPU, PVA v2 visual processing accelerator, 64 GB of 256-bit LPDDR5 memory).

**Figure 12:**
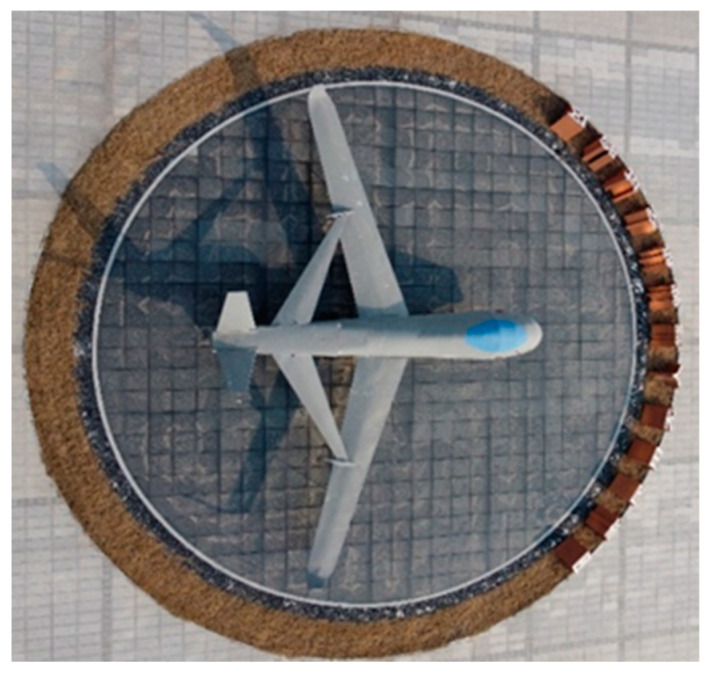
Top view of fixed wing aircraft.

**Figure 13:**
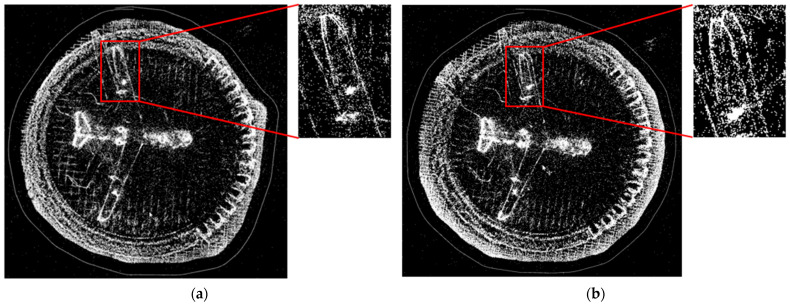
Loop closure detection experiment. (**a**) The loop closure detection module runs; (**b**) the loop closure detection not runs.

In this experiment, we operated the drone to circle around the fixed-wing aircraft shown in Figure 12. By observing whether the semi-dense point clouds constructed at the starting and ending points overlap, we assessed the effectiveness of the loop closure detection module of our algorithm in a real-world setting. Figure 13a and Figure 13b, respectively, represent the semi-dense point cloud maps of the fixed-wing aircraft with and without loop closure detection. The red boxes indicate the semi-dense point cloud maps at the starting and ending points of the drone’s flight. As shown in the red box in Figure 13a, with loop closure detection enabled, the drone successfully triggered loop closure at the starting and ending points, correcting the cumulative trajectory error and allowing the point cloud maps of these points to overlap effectively. In contrast, as illustrated in the red box in Figure 13b, without loop closure detection, the drone could not trigger loop closure at the endpoint, resulting in the inability to eliminate the cumulative trajectory error, which prevented the point cloud maps at the starting and ending points from overlapping. This experiment successfully validated the effectiveness of real-world loop closure detection on the same drone platform.

### 5.2. Driverless Car Experiment

**Figure 14:**
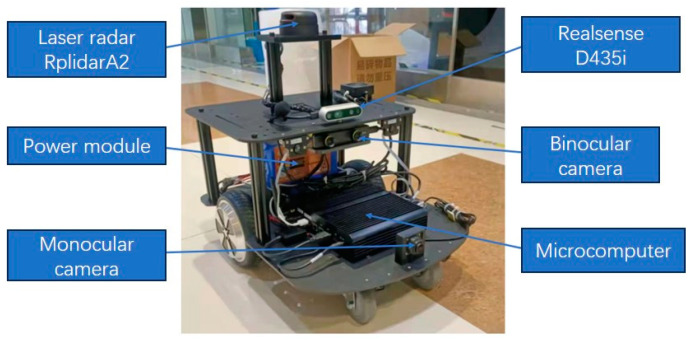
SLAM algorithm test system for unmanned ground platform.

To further verify the effectiveness of the algorithm presented in this paper in handling dynamic objects, we conducted a localization and mapping experiment in an outdoor environment using a ground unmanned platform, as shown in Figure 14 below. To ensure the safety of the experiment, the unmanned platform was placed on the left side of the road, moving forward close to the edge of the road. The image sensor was oriented towards the center of the road to capture dynamic objects on the road. This experiment utilized the monocular mode of the Intel Realsense D435i image sensor mounted on the robot to collect environmental information and the same model of microcomputer as the drone. Due to the limited computing power of the computing platform carried by the unmanned vehicle, the collected image information was sent to a high-performance laptop computer by the unmanned vehicle for instance segmentation and video completion.

**Figure 15:**
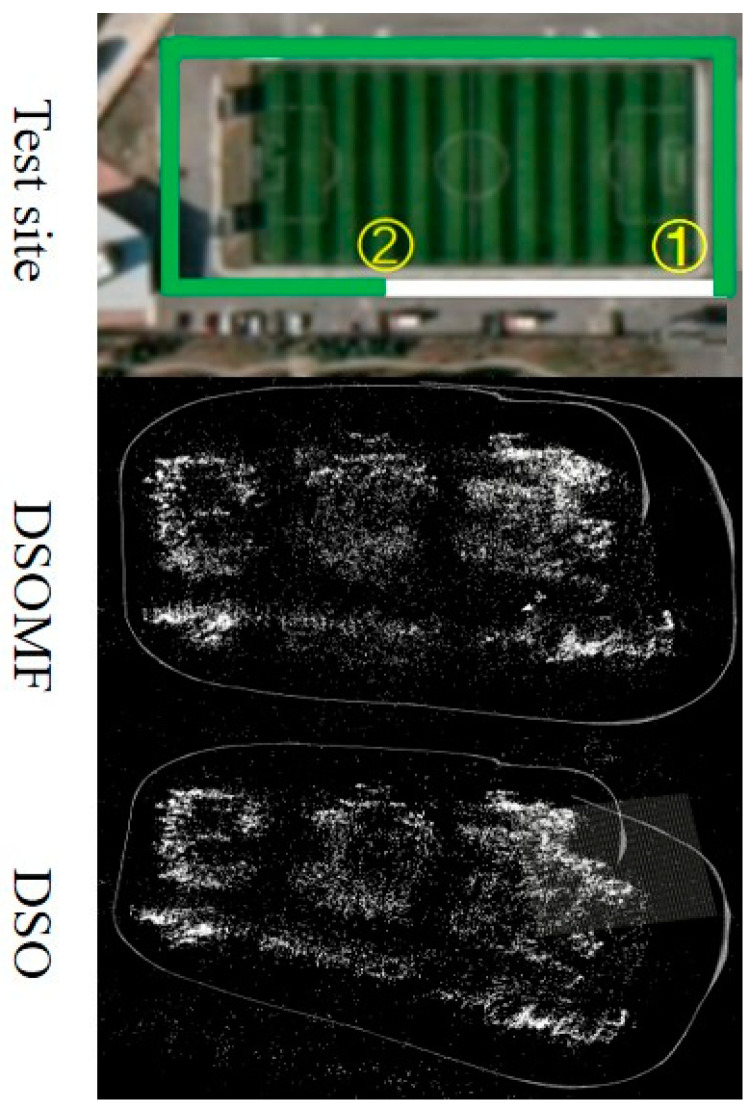
Comparison of outdoor dynamic environment trajectories (In the real-life scenario, the outlined boxes represent the trajectories of dynamic objects. Route one denotes the path of vehicles, while route two signifies pedestrian pathways).

**Figure 16:**
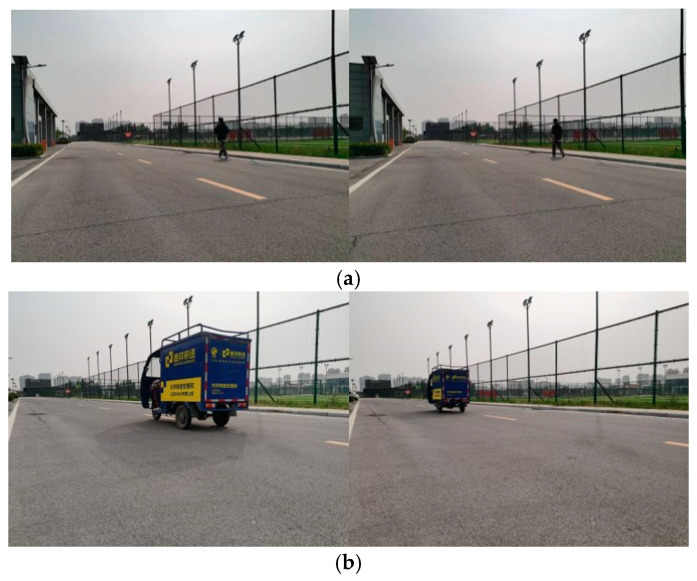
Environment image. (**a**) Pedestrian environment image; (**b**) electric vehicle environment image.

We conducted tests on the algorithm proposed in this study by deploying unmanned vehicles to navigate around the site for one round, using DSO and DSOMF. The trajectories formed using the two algorithms are compared in Figure 15 below. In the site schematic, the green trajectory includes a section where a pedestrian is walking slowly (as shown in Figure 16a), while the white trajectory incorporates a segment with an electric truck moving at a higher speed (as shown in Figure 16b). The comparative observation of the trajectories generated using the two algorithms reveals that the algorithm proposed in this study demonstrates a notable improvement in positioning accuracy in both the white trajectory segments and the sections where loops occur. Upon calculation, it was determined that the processing time per frame for the algorithm discussed in this study was 49.6 milliseconds during the experimental process. Although the dynamic settings of this experiment were somewhat simplified due to hardware limitations, it has nonetheless validated the feasibility of the algorithm presented in this study in real-world scenarios to a certain extent.

## 6. Conclusions

This study addressed the need for high-precision navigation and positioning for unmanned platforms in dynamic environments by introducing instance segmentation and completion into the DSO algorithm framework, proposing a machine learning-based dynamic environment SLAM algorithm. Initially, the algorithm utilizes instance segmentation to divide the scene into objects with potential for motion. Subsequently, it combines semantic and geometric information to identify and eliminate moving objects. After the removal of dynamic objects, a video inpainting algorithm fills in the static background obscured by these moving entities, enhancing the algorithm’s loop closure detection and improving pose alignment accuracy in dynamic environments. Finally, the methodology is validated using the TUM dataset, KITTI dataset, and real-world scenarios. The results demonstrate that in dynamic settings, the positioning accuracy of the proposed algorithm significantly surpasses that of DSO. In our future research, we aim to enhance the real-time performance of the proposed algorithm by improving keyframe selection strategies and data association methods, thereby reducing the computational resource consumption of semantic segmentation and optical flow completion algorithms. Additionally, dynamic objects in the environment hold significant value, and we plan to incorporate them into map construction in our subsequent studies.

## Figures and Tables

**Table 1:** Comparison of absolute trajectory errors in TUM datasets.

Identifier	DSO		Dyna-SLAM		DSOM		DSOMF	
	RMSE	STD	RMSE	STD	RMSE	STD	RMSE	STD
sitting_static	0.009	0.004	0.006	0.003	0.006	0.003	0.006	0.003
walking_static	0.307	0.113	0.037	0.043	0.035	0.039	0.029	0.032
walking_xyz	0.889	0.419	0.091	0.057	0.081	0.047	0.072	0.043

**Table 2:** Execution time and GPU memory cost on KITTI dataset based on several methods.

Algorithm Name	Time (ms/Frame)	GPU Memory Usage/GB
DSO	19	-
DSOM	240	5.2
DSOMF	312	6.5
Dyna-SLAM	300	5.7

**Table 3:** Execution time cost of our method’s main modules.

Algorithm Name	Time (ms/Frame)
Semantic Segmentation	70
Tracking	10
Data Association	5
Image Completion	55

**Table 4:** Comparison of absolute pose errors in KITTI dataset.

Sequence	DSO				DSOMF			
	RMSE	Mean	Max	Min	RMSE	Mean	Max	Min
01	9.478	7.969	16.756	4.125	5.987	6.028	11.763	1.485
02	6.561	5.212	15.608	0.199	5.626	3.697	10.438	0.286
04	1.131	1.759	1.745	0.541	1.067	1.172	2.251	0.318
06	0.886	0.739	1.241	0.433	0.568	0.786	1.081	0.306

**Table 5:** Comparison of absolute pose errors of same-type algorithms.

Sequence	Dyna-SLAM	DSOM	DSOMF
00	3.505	2.937	2.521
01	9.003	7.607	6.420
02	5.219	4.213	4.238
03	1.299	1.054	0.966
04	1.591	1.256	1.214
05	1.779	1.289	1.372
06	0.824	0.672	0.604
07	2.489	2.036	1.880
08	3.291	2.778	2.473
09	2.564	2.101	1.966
10	2.743	2.259	2.375

## Data Availability

The COCO dataset is obtained from http://images.cocodataset.org/zips/train2014.zip (accessed on 6 August 2023). TUM dataset is obtained from https://vision.in.tum.de/data/datasets/rgbd-dataset/download (accessed on 7 August 2023). KITTI dataset is obtained from http://www.cvlibs.net/datasets/kitti/eval_object.php?obj_benchmark=2d (accessed on 9 August 2023). Our real-world test data are available upon request from the corresponding author.
